# Relationship between serum levels of folic acid and homocysteine with cognitive impairment in patients diagnosed with multiple sclerosis

**DOI:** 10.1097/MD.0000000000038680

**Published:** 2024-07-12

**Authors:** Azadeh Imeni Kashan, Zahra Mirzaasgari, Shabnam Nouri Shirazi

**Affiliations:** a School of Medical Sciences, Iran University of Medical Sciences, Tehran, Iran; b Department of Neurology, Iran University of Medical Sciences, Tehran, Iran; c Department of Neurology, Institute for Cognitive Science Studies, Tehran, Iran.

**Keywords:** BICAMS, cognitive impairment, folic acid, homocysteine, multiple sclerosis

## Abstract

Multiple Sclerosis (MS), one of the most common neurological diseases, plays a major role in the ailments of adults. Studies on the role of homocysteine (Hcy) and folic acid in causing cognitive disorders in patients diagnosed with MS are still ongoing. This study aimed to evaluate the serum levels of folic acid and Hcy related to cognitive impairment in patients with multiple sclerosis. This prospective clinical study was conducted on 57 patients diagnosed with MS who were referred to Firoozgar Hospital, Tehran, Iran (Between November 2019 and September 2021). Demographic information and clinical characteristics of enrolled patients were recorded in a predesigned checklist. These characteristics were comprised of outcomes related to the Brief International Cognitive Assessment for MS, and the patient’s Hcy and acid folic levels. Data were analyzed using SPSS version 25. Out of 57 enrolled patients, 39 subjects (68.4%) were female and 18 subjects (31.6%) were male, with a mean age of 36.87 ± 9.40 years old. In terms of disease time span, there was a mean duration of 3.80 ± 4.94 years (range: 1–23 years). There were no significant differences between the mean score of Brief International Cognitive Assessment for MS scale with patient’s sex (*P* value: .88), and disease duration of patients (*P* value: .86). There was no significant relationship between the serum levels of acid folic and Hcy with cognitive impairment (*P* value > .05). The study results revealed that there were no significant relationships between the folic acid, Hcy levels, disease duration, and the type of MS disease with the severity of cognitive impairment. More randomized controlled clinical trials are needed to confirm the relationships between the folic acid and Hcy levels with cognitive impairment in patients with MS.

## 1. Introduction

Multiple Sclerosis (MS), one of the most common neurological diseases, plays a major role in the ailments of adults. In patients diagnosed with MS, demyelination of neuron axons occurs in different parts of the central nervous system. It may appear with a wide range of potential symptoms, including problems related to vision, arm and leg movements, and sensation or balance.^[1–5]^ Based on the Atlas of MS, which is a worldwide open-source in accordance with the MS epidemiology, the prevalence of individuals diagnosed with MS has increased to 2.8 million in 2020, 35.9 per 100,000 people.^[6]^ The exact cause of MS is unknown but the body’s abnormal reactions related to environmental factors are highly considered.^[7–11]^ Previous studies revealed that about half of patients with MS suffered from depression and cognitive disorders. These medical conditions could be induced by the psychological and social burden related to the disease, as well as brain damage, which affects the activity and life quality of patients.^[12,13]^

Recent studies have demonstrated the roles of homocysteine (Hcy), vitamin B12, and folate in patients with MS. It appears that patients diagnosed with MS have higher Hcy levels, and lower vitamin B12 and folate rather than healthy individuals. Folic acid has a significant role in the process of making nucleic acid which is substantial to convert Hcy methionine by methionine synthase enzyme. According to the role of folic acid, its deficiency may lead to an increase in the serum level of Hcy. Based on some previous studies, an increase in Hcy levels has been reported in MS and other cognitive diseases, like Parkinson disease and dementia.^[14–20]^

However, studies on the role of Hcy and folic acid in causing cognitive disorders in patients diagnosed with MS are still ongoing. This study aimed to evaluate the serum levels of folic acid and Hcy related to cognitive impairment in patients with MS.

## 2. Methods

This prospective clinical study was conducted on 57 patients, diagnosed, and confirmed with MS, who were referred to the Firoozgar Hospital, Tehran, Iran (between November 2019 and September 2021). This study was approved by the research deputy of Iran University of Medical Sciences, Tehran, Iran (Code: IR.IUMS.FMD.REC.1399.032). Written and Informed consent was obtained from all included patients.

In this study, all patients were diagnosed and confirmed with MS by an expert neurologist based on McDonald diagnostic criteria for MS.^[21]^ Demographic information and clinical characteristics of enrolled patients were recorded in a predesigned checklist. Clinical characteristics comprised of outcomes related to the Brief International Cognitive Assessment for Multiple Sclerosis (BICAMS), and the patient’s Hcy and acid folic levels. The BICAMS test, which is First Consensus Steps Towards a Brief Universal Cognitive Assessment for MS, is composed of 3 parts: The Symbol Digit Modalities Test (SDMT), The California Verbal Learning Test–II (CVLT-II), and The Brief Visuospatial Memory Test-Revised (BVMT-R). The SDMT19 is recommended as the test of information processing speed. The test consists of single digits paired with abstract symbols. The patient must say the number that corresponds with each symbol. The SDMT can be completed within 5 minutes, including instructions, practice, and testing. The CVLT-II test is planned to assess verbal memory (immediate recall). This test comprises a 16-item word list, with 4 items belonging to each of the 4 categories, arranged randomly. Patients are required to recall as many items as possible, in any order, after each reading of the list. The CVLT-II T1–5 can be completed in 5 to 10 minutes, including instructions, testing, and responses. The BVMT-R is validated to assess visual memory (immediate recall). The BVMT-R test requires the patient to inspect a 2 × 3 stimulus array of abstract geometric figures. The array is removed and the patient is required to draw the array from memory, with the correct shapes in the correct position.

### 2.1. Data collection and analyses

In this study, 5 mL of blood was taken from the cubital vein of eligible patients and transferred into yellow-capped serum tubes. After that, blood was centrifuged (1500 × g for 10 minutes at +40°C). The serum was separated and stored in Eppendorf tubes (−80°C) before analysis. Serum levels of Hcy and folic acid were measured after taking blood from the enrolled patient using related laboratory kits. Finally, the diagnostic test and laboratory results of Hcy and folic acid serum levels were analyzed using SPSS 25 statistical tests. *P* value ≤ .05 was considered significant.

## 3. Results

In this study, out of 57 enrolled patients diagnosed with MS, 39 subjects (68.4%) were female and 18 subjects (31.6%) were male, with a mean age of 36.87 ± 9.40 years old. In terms of disease time span, there was a mean duration of 3.80 ± 4.94 years (range: 1–23 years). There was no significant difference between the patient’s sex and their disease duration (*P* value > .05). In addition, regarding the MS types, the frequency of relapsing-remitting MS, primary progressive MS, and secondary progressive multiple sclerosis (SPMS) was reported as 36 subjects (63.2%), 16 subjects (28.1%), and 5 subjects (8.8%), respectively.

In this study, out of 57 enrolled patients, 10 patients (17.50%) with cognitive impairment, and 47 patients (82.5%) with no cognitive impairment were reported. The mean score of the BICAMS scale was reported as −1.05 ± 0.24 (range: −2.20 to 1.70). The mean score of the BICAMS scale in females and males was reported as −1.06 ± 0.23, and −1.04 ± 0.27, respectively (Table 1). There were no significant differences between the mean score of BICAMS scale with patient’s sex (*P* value: .88), and disease duration of patients (*P* value: .86).

**Table 1:** The mean score of BICAMS scale based on patient’s sex.

BICAMS score		Min	Max	Mean ± SD	*P* value
Patient’s sex	Male	−2.20	1.10	−1.04 ± 0.27	.88
	Female	−2.10	1.70	−1.06 ± 0.23	
Duration	Male	1	12	3.05 ± 5.05	.86
	Female	1	23	4.14 ± 4.89	

BICAMS = Brief International Cognitive Assessment for multiple sclerosis, SD = standard deviation.

In accordance with the level of folic acid, the mean serum level of folic acid in all enrolled subjects was reported as 7.66 ± 4.91 mcmol/L (range: 1.30–20 mcmol/L). The mean serum level of folic acid in males and females was reported as 8.13 ± 4.90, and 6.65 ± 4.93 mcmol/L. There was no significant difference between the mean serum level of folic acid and the patient’s sex (*P* value: .29). Based on the results, out of 57 patients enrolled in the present study, 33 subjects (57.9%) were reported with a normal range, and 24 subjects (42.1%) were presented with an abnormal range (folate deficiency). The Mean ± SD of folic acid serum level was reported in Table 2, in detail.

**Table 2:** The mean serum level of folic acid and homocysteine based on patient’s sex.

		N (%)	Min	Max	Mean ± SD	*P* value
Folic acid	Male	18 (31.6)	1.30	20	4.93 ± 6.65	.29
	Female	39 (68.4)	2.20	20	4.90 ± 8.13	
	Total	57 (100)	1.30	20	4.91 ± 7.66	
Homocysteine	Male	18 (31.6)	4	31	8.25 ± 14.06	.34
	Female	39 (68.4)	2.20	36	9.30 ± 16.51	
	Total	57 (100)	2.20	36	8.98 ± 15.74	

In terms of serum Hcy levels, the Mean ± SD of Hcy serum level was reported as 15.74 ± 8.98 mcmol/L (range: 2.20–20 mcmol/L). Based on the results, out of 57 patients enrolled in the study, 25 cases (43.90%) were presented with a normal range, and 32 cases (56.10 %) were reported with an abnormal range. The Mean ± SD of Hcy serum level was reported in Table 2, in detail. The mean serum level of Hcy in females and males was presented as 16.51 ± 9.30, and 14.06 ± 8.25, respectively. There was no significant difference reported between the Hcy serum levels and the patient’s sex (*P* value: .34), whereas, the Mean ± SD of Hcy serum levels was greater in females (16.51 ± 9.30) than in males (14.06 ± 8.25).

Based on the results, there was no significant relationship between the serum levels of folic acid and Hcy with patient’s cognitive impairment (*P* value > .05). Moreover, there was no significant association between the serum levels of Hcy and acid folic with the MS types (*P* value: .78; Table 3).

**Table 3:** The mean serum level of folic acid and homocysteine based on MS types.

		Min	Max	Mean ± SD	*P* value
Folic acid	RRMS	1.30	20	5.10 ± 7.62	.61
	PPMS	3	20	4.04 ± 7.81	
	SPMS	3.80	20	7.0 ± 7.40	
Homocysteine	RRMS	4	36	8.84 ± 14.85	.07
	PPMS	6	31	8.91 ± 19.51	
	SPMS	2.20	17.50	8.98 ± 15.74	

MS = multiple sclerosis, PPMS = primary progressive multiple sclerosis, RRMS = relapsing-remitting multiple sclerosis, SPMS = secondary progressive multiple sclerosis.

## 4. Discussion

MS, which is known as a chronic disease with inflammation and neurodegeneration, has a crucial role in adult ailments. In patients diagnosed with MS, demyelination of neuron axons occurs in different parts of the central nervous system. It may cause severe progressive disabilities. Cognitive dysfunction and spasticity are defined as an indication of disease progression.^[1–5]^ Recent studies have demonstrated the roles of Hcy, B12 vitamin, and folate in patients with MS. It appears that patients diagnosed with MS and other cognitive diseases, like Parkinson disease and dementia, have higher Hcy levels, lower vitamin B12 and folate rather than healthy individuals.^[14–20]^

Based on previous epidemiological studies, a decrease in serum level of folate and vitamin B12, and hyperhomocysteinemia associated with cognitive impairment was reported.^[22–26]^ A study performed by Ma et al^[27]^ on plasma Hcy and serum folate and vitamin B12 levels in mild cognitive impairment (MCI) and Alzheimer disease (AD) demonstrated that in older adults, low blood levels of folate and vitamin B12 and elevated Hcy levels were associated with MCI and AD, and the association was stronger for AD.

In another study carried out by Kong et al, cognitive impairment was associated with hyperhomocysteinemia in middle-aged and elderly people in China. Based on the mentioned study, serum Hcy concentration was negatively associated with total basic cognitive aptitude tests score, which applied to evaluate the cognitive function.^[28]^

A study by Morris et al^[29]^ indicated a faster rate of cognitive decline was related to high folate intake from food. On the contrary, high total B12 intake was associated with slower cognitive decline among the oldest subjects. Furthermore, a decrease in Hcy levels has been observed in AD patients in a trial that used a combination of folic acid, vitamin B12, and vitamin B6.^[30]^ In addition, a community-based study revealed that the higher serum level of Hcy was associated with a risk of AD, and higher B12 and folate levels were protective parameters to AD.^[31]^

Hcy might cause its harmful effects on the nervous system by triggering the *N*-methyl-d-aspartate receptor, which can lead to the death of cells, or by transforming into homocysteic acid, which can also negatively impact neurons. The link between low levels of folate and vitamin B12 and cognitive disease could be due to the influence these vitamins have on Hcy levels, or it could be tied to their role in brain methylation reactions.^[32–35]^

In a study conducted by McMahon and colleagues, 276 participants with hyperhomocysteinemia, who underwent folate therapy, were evaluated, and followed up for 2 years. Based on the mentioned study results, despite a significant decrease in Hcy levels, no significant difference was reported in patients regarding cognitive impairment between the 2 study groups.^[36]^ Besler and Comoğlu^[37]^ performed a study to measure the folic acid and Hcy levels in 24 patients with MS and 20 healthy individuals. The study’s results demonstrated that the serum level of Hcy in patients with a low level of folate and vitamin B12 was significantly higher but there was no significant difference between the study groups. The mentioned study concluded that there is no significant relationship between the serum level of Hcy and folic acid. Another study conducted by Ramsaransing et al^[38]^ reported that there was no significant relationship between Hcy and folate levels. The present study results were consistent with the mentioned studies. In the present study, despite the high frequency of hyperhomocysteinemia, no significant relationship was reported between the level of Hcy and cognitive impairment. In this regard, our findings are in contrast with some previous studies.^[19,39]^ These studies revealed that there is a substantial relationship between hyperhomocysteinemia in patients with MS and their cognitive impairment. Besides, some recent studies have presented a positive relationship between hyperhomocysteinemia and the occurrence of dementia.^[18,39–41]^

The link between cognitive impairment diseases and Hcy levels remains a topic of debate. As mentioned before, some research has indicated a rise in Hcy levels in people with MCI, while other studies have not found this to be the case. These inconsistent results could potentially be attributed to the different methods and criteria used to diagnose cognitive impairment, and the degree of cognitive impairment.

This study has some limitations. The short duration of patient’s follow-up is considered one of the limitations. In addition, the small number of enrolled patients, and low duration of disease among enrolled patients considered other limitations of the present study. In this study, we include all eligible patients who were diagnosed and confirmed with MS by an expert neurologist based on McDonald diagnostic criteria for MS.

## 5. Conclusion

The study results revealed that there were no significant relationships between the blood folate concentration, Hcy levels, disease duration, and the type of MS disease with the severity of cognitive impairment. More randomized controlled clinical trials are needed to confirm the relationships between the folic acid and Hcy levels with cognitive impairment in patients with MS.

## Author contributions

**Formal analysis:** Shabnam Nouri Shirazi.

**Methodology:** Shabnam Nouri Shirazi.

**Project administration:** Azadeh Imeni Kashan.

**Supervision:** Zahra Mizaasgari.

**Writing – original draft:** Azadeh Imeni Kashan.

**Writing – review & editing:** Azadeh Imeni Kashan.

**Concept and design:** Azadeh Imeni Kashan, Zahra Mirzaasgari.

**Acquisition, analysis, or interruption of data:** Shabnam Nouri Shirazi, Azadeh Imeni Kashan.

**Drafting of the manuscript:** Azadeh Imeni Kashan.

**Critical revision of the manuscript for important intellectual content:** Azadeh Imeni Kashan, Zahra Mirzaasgari.

**Statistical analysis:** Shabnam Nouri Shirazi.

**Administrative, technical, or material support:** Azadeh Imeni Kashan.

## Correction

This article was originally published with author name Azadeh Imeni Kashan spelt incorrectly as Azadeh Imani Kashan. It has now been corrected in the online version.
